# Connaissances et comportements au regard de la santé reproductive: enquête chez les marocains en zone rurale

**DOI:** 10.11604/pamj.2016.25.186.9940

**Published:** 2016-11-24

**Authors:** Majda Sebbani, Latifa Adarmouch, Isam Azzahiri, Wafa Quiddi, Mohamed Cherkaoui, Mohamed Amine

**Affiliations:** 1 Service de Recherche Clinique, CHU Mohammed VI de Marrakech, Département de Médecine Communautaire et de Santé Publique, Faculté de Médecine et de Pharmacie de Marrakech, Université Cadi Ayyad, Maroc; 2 Laboratoire d’Ecologie Humaine, Département de Biologie, Faculté de Sciences, Université Cadi Ayyad de Marrakech, Maroc

**Keywords:** Santé reproductive, connaissances, attitudes, pratiques, homme, Maroc, Reproductive health, knowledge, attitudes, practices, man, Morocco

## Abstract

**Introduction:**

Décrire les connaissances, attitudes et pratiques (CAP) des hommes d'une population enclavée à l'égard de la santé reproductive.

**Méthodes:**

Il s'agissait d'une étude mixte (focus group et étude CAP) menée dans une région montagneuse prés de Marrakech. L'enquête descriptive a recruté 74 hommes par deux méthodes d'échantillonnage (visiteurs du souk hebdomadaire et accompagnateurs à une compagne sanitaire). La collecte des données (Mars - Avril 2014) était par questionnaire traduit en dialectale et administré par des enquêteurs formés parlant Amazigh. L'analyse des données quantitatives était descriptive et bivariée (seuil de signification statistique à 5%). Le focus group était analysé après enregistrement et retranscription.

**Résultats:**

La médiane de l'âge était de 38 ans [19 à 80 ans]. Parmi 64 hommes vivant en couple (86,5%), la moitié (55,4%) optait pour la pilule. Les 2 tiers ont reconnu l'intérêt de surveiller la grossesse, tandis que 37% des maris ignoraient la fréquence du suivi. Le non recours à la consultation prénatale était de 56,9% (N=58), dont 42,4% sans raison. Le niveau d'instruction était associé au suivi de la grossesse par la conjointe (p=0,015) et à l'attitude positive à l'égard de son intérêt (p=0,011). La méconnaissance de la possibilité de complications (35,1% des répondants) était un facteur de non recours à la consultation post-natale par la conjointe (p=0,021). Les barrières relevées de l'analyse du focus group étaient d'ordre culturel, géographique et socioéconomique.

**Conclusion:**

Des actions d'information, d'éducation et de communication devraient viser d'avantage l'implication du sexe masculin dans des contextes similaires.

## Abstract (en)

**Introduction:**

The objective is to describe the knowledge, attitudes and practices (KAP) of men living in a landlocked area towards reproductive health.

**Methods:**

We conducted a mixed methods study (focus group and KAP survey ) in a mountainous region near Marrakech. This descriptive survey involved 74 men ; we used two sampling methods (souk visitors on a weekly basis and persons accompanying the expectant mothers to their antenatal visits as part of an antenatal clinic caravan campaign). Data collection (March - April 2014) was made using a questionnaire translated into respondents' dialect and administered by trained Amazigh-speaking investigators. Quantitative data analysis was descriptive and bivariate (statistical significance level at 5%). Focus group data were analyzed after recording and transcription.

**Results:**

The median age was 38 years (19-80 years). Of the 64 men living together (86.5%), half (55.4%) opted for the pill. Two thirds recognized the importance of importance of pregnancy monitoring, while 37% of husbands were unaware of the frequency of follow-up visits. The non-use of services was 56.9% (N = 58) of which 42.4% without any reason. Educational attainment was associated with spouse's pregnancy screening (p = 0.015) and with a positive attitude toward the importance of pregnancy monitoring (p = 0.011). The lack of awareness regarding possible complications (35.1% of respondents) was a factor associated with lack of postnatal care among spouses (p = 0.021). The barriers identified in focus group analysis were cultural, geographic and socio-economic.

**Conclusion:**

Information, education, communication programmes should mostly aim to involve male participants in similar contexts.

## Introduction

Le Maroc, à l'instar des autres pays; s'est engagé dans les politiques qui visent à atteindre le 5^ème^ objectif du millénaire concernant le développement pour l'amélioration de la santé maternelle. Dans la dernière enquête nationale sur la santé familiale, le ratio de mortalité maternelle est de 112 pour 100000 naissances vivantes ce qui témoigne des progrès réalisés dans la réduction de la mortalité maternelle. Cependant de grandes disparités persistent entre milieu urbain et rural (73 versus 148/10000 naissances vivantes) [1]. Le taux d'accouchement en milieu surveillé en 2011 était de 72,7% avec prés de 54,6% dans le milieu rural (90,7% en urbain). La proportion des femmes ayant bénéficié des consultations prénatales chez un personnel qualifié est de l'ordre de 77,1% (contre 67,8 % en 2004). Cette proportion diffère significativement du milieu urbain au milieu rural soit 91,6% contre 62,7% [2]. En vue de consolider les acquis et de renforcer les actions entreprises, le ministère de la santé a élaboré la stratégie nationale d'accélération de la réduction de la mortalité maternelle 2012 - 2016. Elle vise à maintenir un taux de prévalence contraceptive supérieur ou égal à 67%; à augmenter la couverture des accouchements en milieu surveillé de 73% à 90% (de 55% à 75% pour le milieu rural); à augmenter la couverture en consultation prénatale de 77% à 90%; et de 95% pour la consultation du post-partum [3]. La mobilisation sociale à travers le renforcement de la participation communautaire dans la promotion de la maternité sans risques et de la santé de la reproduction figure parmi les axes d'intervention majeurs [4]. L'implication de la population est une pierre angulaire pour la réussite de tels programmes surtout en milieu rural. Le concept de la santé de la reproduction dans une perspective plus large que la “santé maternelle et infantile”, inclut la santé sexuelle et la régulation de la fécondité. Il dépasse le strict cadre médical pour poser la question de la responsabilité individuelle et collective dans les comportements sexuels et reproductifs. De cette évolution conceptuelle découle l'implication des hommes qui reste néanmoins en grand décalage avec la réalité et les représentations sociales dans plusieurs cultures et pays du monde [5].

Le questionnement sur la place des hommes dans la santé sexuelle et reproductive ne peut être réfuté. Ses comportements sont importants à analyser car ils peuvent avoir un lien avec la morbi - mortalité maternelle et infantile surtout lorsqu'il s'agit d'une population où les modèles de pensées masculines prédominent. Mais leurs rôles est souvent négligé en face de la responsabilisation des femmes [6, 7]. Plusieurs pays ont saisi l'intérêt de cibler davantage les hommes lors des actions d'éducation et d'information autour des signes de complications de la grossesse [8]. Les facteurs liés au mari tels que le niveau d'instruction ou le niveau de connaissances des signes de risques de la grossesse sont associés à l'utilisation des soins prénatals de la femme [9, 10]. L'attitude positive des hommes et leur présence aux consultations prénatales peut conduire la conjointe à l'utilisation des services de soins durant sa grossesse [11]. Cela témoigne de l'impact majeur de la participation masculine dans la gestion de la grossesse par le couple et sur l'adhésion de la femme aux programmes de surveillance et de suivi en pré et postnatal. Comprendre les pensées et les comportements des hommes est essentiel pour mieux viser les actions à entreprendre dans le cadre de la promotion de la santé maternelle et reproductive. Des études qui donnent la parole aux hommes dans ce contexte sont peu nombreuses. L'objectif de l'étude était de décrire les connaissances, attitudes et pratiques des hommes d'une région rurale au regard de la santé reproductive et maternelle.

## Méthodes

Il s'agissait d'une enquête observationnelle transversale de type connaissances, attitudes et pratiques réalisée en population générale auprès des hommes habitants dans une commune rurale située à 78 km de la ville de Marrakech dans le sud marocain. Elle était complétée par un focus group. Le lieu de l'étude est une zone appartenant à la chaine du grand atlas, caractérisée par ses conditions climatiques difficiles et sa faible accessibilité malgré sa proximité géographique de la ville. Elle est peu desservie en structure de soins (un seul centre de santé) et connait une grande précarité. La population enquêtée était représentée par les hommes adultes. L'échantillon était composé à partir de deux méthodes: a) accidentel à partir des accompagnateurs des femmes enceintes venant à la consultation prénatale dans le cadre d'une caravane sanitaire organisée en parallèle à la même étude; b) à partir de la visite du souk hebdomadaire ayant lieu chaque dimanche dans la commune. C'est une méthode d'échantillonnage non probabiliste inspirée par l'expérience d'Immpact pour la mesure de la mortalité maternelle mais qui est représentative de la population enquêtée. Étant donné l'habitat dispersé, le tirage aléatoire n'est pas possible.

En milieu rural, les personnes se rendant au marché local sont considérés comme représentatives des membres de leurs communautés [12]. L'échantillon final était constitué des hommes ayant accepté la participation à l'enquête. La collecte des données s'est déroulée durant les mois de mars et avril 2014. Les données quantitatives ont été collectées par méthode d'entretien en face à face à l'aide d'un questionnaire administré par des enquêteurs (étudiants en médecine) formés et parlant Amazigh vu la spécificité culturelle de la région. L'outil en arabe dialectal marocain était composé des sections suivantes: a) connaissances, attitudes et pratiques concernant la planification familiale, le déroulement et la surveillance de la grossesse et de l'accouchement, et la période du post-partum; b) caractéristiques sociodémographiques: âge, situation familiale et économique, couverture sociale; **c)** accessibilité et recours aux soins.

Le focus groupe s'est déroulé en présence d'un enquêteur et d'un observateur auprès de quatre hommes ayant accepté d'être interviewés et enregistrés parmi 10 hommes sollicités à participer. Le guide ayant servi à mener l'entretien a été élaboré autour de quatre thèmes. Les participants ont été invités a échangé durant 45 min à propos des questions posées, les relances ont permis de réorienter le discours et d'explorer les représentations et perceptions à l'égard de la participation des hommes dans la planification des naissances et la gestion de la grossesse et de l'accouchent ainsi que les différents barrières ressenties.

Les analyses de type descriptif et bivarié ont été réalisées par le logiciel SPSS version 16. Les variables qualitatives ont été décrites par les effectifs et les pourcentages correspondants. Les variables quantitatives par les mesures de tendances centrales (médiane) et les mesures de dispersion (étendue). Le test de Khi Carré était utilisé pour comparer les proportions. Le seuil de signification statistique était de 5%. L'entretien était retranscrit mot par mot pour l'analyse thématique du contenu. Sur le plan réglementaire, les autorisations des autorités sanitaires locales ont été obtenues. Tous les participants ont été informés des objectifs de l'étude, et leur consentement oral a été obtenu avant de démarrer l'enquête. L'anonymat et la confidentialité des données ont été assurés.

## Résultats

### Caractéristiques sociodémographiques des participants

La médiane de l'âge était de 38 ans (étendue de 19 à 80 ans). La majorité des participants était mariés (86,5%) et exerçait une profession (89,2%), dont la moitié des cas dans la même commune. Prés de 40,5% étaient scolarisés. Les modes de vie prédominants étaient dans 46% des cas représentés par la famille nucléaire et dans les mêmes proportions avec les parents. En cas de problème de santé, les deux tiers des participants préféraient consulter une autre structure que le centre de santé local. Les principales raisons du non recours étaient l'insatisfaction ou le manque de personnel. Les autres caractéristiques d'accès et de recours aux soins sont décrites dans le Tableau 1.

**Tableau 1:** Caractéristiques d’accès aux soins des participants à l’étude

Les caractéristiques	Effectif (n)	Pourcentage (%)
**Couverture sanitaire**	16	21,6
**Moyen pour arriver au centre de santé le plus proche**		
A pieds	39	52,7
Transport commun	35	47,3
**Temps pour y arriver**		
≤ 30 min	31	41,9
30 min à 2 heures	31	41,9
Plus de 2 heurs	12	16,2
**Recours au centre de santé en cas de problème de santé**	25	33,8

### Résultats de l'enquête connaissances, attitudes, et pratiques

**Planification des grossesses**: Près de 77% des hommes ont rapporté connaître au moins un moyen de contraception. Parmi les méthodes citées, la pilule vient en premier dans toutes les réponses. Le préservatif ne l'était que dans 21,8% des cas. La principale source d'informations était l'entourage de l'enquêté (62%), suivi des professionnels de santé et de la télévision (13,5% et 9,5% respectivement). Le nombre désiré d'enfants était de 2 à3 dans 60,8% des réponses. Parmi 64 hommes vivant en couple, uniquement la moitié (55,4%) utilisait une contraception (pilule dans 100% des cas) (Tableau 2).

**Tableau 2:** Connaissances, attitudes et pratiques des participants vis-à-vis de la planification familiale

CAP ^a^ à l’égard de la planification familiale	Effectif (n)	Pourcentage (%)
**Accord pour le contrôle des naissances**	48	65,0
**Nombre idéal d’enfants**		
2 à 3	45	60,8
4	18	24,3
5 ou plus	05	06,8
Je ne sais pas	06	08,1
**Moyens de contraception connus**		
Pilule seule	33	44,6
Pilule, ligature des trompes et préservatif	10	13,5
Pilule et dispositif intra-utérin	07	09,5
Pilule et injectable	03	04,0
Pilule et préservatif	02	02,7
Aucun	18	24,3

a CAP : **C**onnaissances, **A**ttitudes et **P**ratiques

**Grossesse, accouchement et postpartum**: Les deux tiers des interrogés ont reconnu la nécessité de surveiller la grossesse par un professionnel de santé. Plus que la moitié de ceux qui vivaient en couple (N=64) a répondu connaître le nombre de consultations prénatals à effectuer durant la grossesse, alors que 37,5% ont répondu méconnaître la fréquence et le rythme du suivi. La fréquence de trois consultations était rapportée par un tiers des participants (32,5%). Parmi 23 interviewés ayant assisté à une complication au cours de la grossesse dans leur entourage, la conduite jugée adaptée dans 4 situations était celle de garder la femme à domicile. Le non recours à la consultation prénatale par le couple était noté dans 56,9% des cas (N = 58), dont 42,4% sans aucune raison avancée. Quand à l'accouchement et la période postnatale, 59,5% des répondants préféraient l'accouchement à domicile. La gratuité, la proximité et le soutien de l'entourage étaient les principales motivations. Le dernier accouchement n'était surveillé par un professionnel de santé que dans 26,2% des cas (N=55). Un tiers des participants méconnaissaient la possibilité de complications chez la femme en post-partum (36%) et 17% ont jugé qu'il n'est pas nécessaire d'effectuer la consultation post-natale. Chez les 32% ayant eu recours aux structures de soins en postnatal, la vaccination du nouveau-né était le motif principal (58,3%) (Tableau 3 et Tableau 4).

**Tableau 3:** Recours aux consultations pré et post-natales

Variables de recours aux soins pré et post-natales	Effectif (n)	Pourcentage (%)
**Raisons de non recours à la consultation prénatale (N=33)**		
Sans raisons	14	42,4
Inaccessibilité géographique	06	18,2
Structure de soins insatisfaisante ou absente	07	21,2
Inaccessibilité financière	04	12,1
Mari occupé	02	06,1
**Raisons du recours à la consultation postnatale (N=24)**		
Vaccination du nouveau-né	14	58,3
Simple contrôle	06	25,0
Complications chez la mère	04	16,7

**Tableau 4:** Raisons de non surveillance de l’accouchement (N=38)

Les motifs exprimés	Effectif (n)	Pourcentage (%)
Absence de complications	21	55,3
Eloignement des structures d’accouchement	12	31,6
Inaccessibilité financière	10	26,3
Manque de confiance	03	07,9
Présence d’autres enfants à garder	02	05,3

**Facteurs associés aux CAP**: Concernant les facteurs déterminants les connaissances, attitudes et comportements des participants, le niveau d'instruction des hommes était significativement associé au suivi de la grossesse par la conjointe (p=0,015) et à l'attitude positive à l'égard de l'intérêt de surveiller la grossesse par un professionnel de santé (p=0,011). En postpartum, la méconnaissance de la possibilité de complications (36% des répondants) était associée au non recours à la consultation post-natale par la conjointe (p=0,021).

### Synthèse du focus group

L'analyse du discours des interviewés a permis d'explorer les représentations et perceptions des hommes vis à vis de la planification des naissances, de la surveillance de la grossesse en pré et postnatale. Il ressortait de cette analyse les points suivants: a) la planification familiale n'est pas l'affaire de l'homme, ce dernier exprime le désir d'avoir des enfants rapidement après le mariage. *« On ne planifie pas, la grossesse arrive naturellement » « il faut que tu te marie pour avoir des enfants tant que tu es encore jeune », « …pour que les enfants grandissent et m'apportent de l'aide dans les charges de la vie ! »* ; b) la grossesse est une période de la vie génésique d'une femme, et non pas une maladie pour aller consulter. Cette période ne doit pas apporter un changement aux habitudes de la femme rurale, elle doit continuer à exercer ses taches ménagères et s'occuper de ses enfants jusqu'à l'accouchement. *« La femme doit accoucher à la maison… », « Pourquoi consulter !, elle est enceinte et non malade… », « Il faut surveiller la grossesse, mais ici on ne le fait pas… »* ; c) les hommes ont exprimé l'intérêt de la surveillance de la grossesse malgré le manque d'informations et de connaissances relevé: *« Il faut surveiller la grossesse, mais ici on ne le fait pas », « les femmes surveillent la grossesse une fois par semaine : on leur mesure le poids et la hauteur de l'abdomen, c'est ça ! » «Ici, il n'y a pas assez de sensibilisation… »* ; d) les freins relevés sont d'ordre culturel, et socio- économique en plus de l'insatisfaction à l'égard des prestations de soins : *« Les femmes ici préfèrent la maison… » « La femme est entouré chez elle, on lui prépare un bon repas chaud, et le bébé ira bien, pourquoi aller à l'hôpital, on n'a pas d'argent !» « La femme consulte quand elle a des contractions ou complications… », « …ici, on n'a pas de médecin, ni hôpital ni médicament, on doit partir à la ville la plus proche… »*

## Discussion

La prise de décision, l'adoption d'une méthode contraceptive et la poursuite de son utilisation par la conjointe sont influencées par la position de l'homme. Or, depuis l'avènement de la pilule, les politiques de santé et les stratégies de planification familiale ciblent davantage la femme dans la promotion de la santé reproductive. Alors que l'homme garde une influence sur la gestion de la dynamique contraceptive du couple. Le nombre idéal d'enfants désiré par nos participants était faible dans la majorité des réponses (moins de 3 dans 60,8% des réponses), ce qui témoigne d'une certaine évolution des conceptions. Cependant la proportion des enquêtés qui acceptaient l'utilisation de moyens contraceptifs était de 65%, chiffre plus faible que la prévalence national de 94% selon la dernière enquête nationale de 2011 [2].

Malgré l'existence de méthodes variées, la pilule était celle la plus connue chez notre échantillon contrairement au préservatif qui n'a été cité que dans 21,8%. Ce constat diffère de celui décrit dans la littérature (98% des hommes rapportent le préservatif) [13]. Il peut être attribué d'une part à la gratuité de la pilule et sa disponibilité dans les centres de santé marocains dans le cadre du programme de planification familiale. Ce qui explique que cette méthode reste adoptée par tous nos enquêtés qui utilisaient un moyen contraceptif (55,4% des hommes mariés). D'autre part, il est expliqué par le manque de sensibilisation des hommes dont les connaissances sont largement influencées par l'entourage; source d'information dans 62% des réponses.

Dans une revue de la littérature, le concept de « la planification familiale » était bien connu des hommes dans des pays à contexte similaire. Au Niger par exemple, 99% des hommes enquêtés étaient au courant de l´existence d'au moins deux contraceptifs modernes. Chiffre plus élevé que celui retrouvé dans notre cas (77%). Dans une autre étude menée en Ethiopie, 98% des hommes interrogés (parmi 811 couples) ont soutenu l´utilisation des méthodes de planification familiale, mais leurs pratiques étaient très faibles. Il existe un désaccord entre les connaissances et les pratiques des hommes concernant ce point [13, 14]. Ce qui rejoint les résultats de notre étude, vu que seulement la moitié des mariés pratiquaient une contraception.

Ces résultats sont dus à la persistance des barrières culturelles en plus du manque d'information et de sensibilisation ciblée sur la participation masculine dans la planification familiale, surtout dans un contexte de pauvreté et d'analphabétisme où donner naissance à un enfant est considéré comme un signe de réussite du mariage et comme une éventuelle ressource pour la famille : *« Il faut que tu te marie pour avoir des enfants tant que tu es encore jeune », « …pour que les enfants grandissent et m'apportent de l'aide dans les charges de la vie ! »*. Les aspects relatifs à la grossesse et à l'accouchement sont également vus comme une affaire purement féminine chez les populations du monde rural dans les pays en développement. L'étude menée par Signth et al. en Uganda illustre le modèle de pensée des hommes et de la société [15]. Les intervenants insistent sur l'importance d'évaluer les connaissances et les croyances des hommes lors de toute approche qui vise l'amélioration de la santé maternelle. Il est primordial de prendre conscience de l'intérêt de la surveillance de la grossesse, de connaître les singes de complications de celle-ci et de promouvoir le recours à l'accouchement surveillé par un professionnel de santé chez les populations en zone rurale où analphabétisme et accès difficile aux soins cohabitent [16, 17].

Dans une étude menée dans une zone rurale au Bangladesh, seul 27% des enquêtés (N=480) accompagnaient leurs femmes à la consultation prénatale [17]. Les 2 tiers de nos participants ont reconnu la nécessité de surveiller la grossesse par un professionnel de santé. Mais les pratiques étaient contradictoires chez la moitié des hommes mariés. Le non recours était inexpliqué dans plus de 40% des réponses. Alors que Les principales raisons sont représentées par les difficultés d'accès géographiques et financières et l'offre de soins absente ou insatisfaisante. En effet l'absence de couverture sociale (78,4%) et la pauvreté qui caractérise la région sont des facteurs d'inaccessibilité aux soins courants. Uniquement 33% des répondants recourent au centre de santé en cas de problème. Le facteur économique est décrit dans la littérature. En Malawi, la plupart des maris enquêtés par Aarnio et al. se sentent responsables de la préparation à la naissance, mais la faible disponibilité et les coûts de transport pourraient empêcher le recours aux soins même devant des signes de complications de la grossesse [18]. En plus, la région enquêtée est à caractère montagnard, les ménages sont dispersés géographiquement et la commune n'est desservie que par un seul centre de santé. Ce qui entrave l'accès de la population surtout en hiver où les routes deviennent impraticables à cause de la neige.

D'autres facteurs pourraient être déterminants des croyances et comportements du mari comme le niveau d'instruction [19]. Dans notre étude il était significativement associé au suivi de la grossesse par la conjointe (p = 0,015) et à l'attitude positive à l'égard de l'intérêt de surveiller la grossesse par un professionnel de santé (p = 0,011). Près de 46% des interrogés vivaient avec leurs parents ce qui pourraient avoir un impact sur les comportements des couples. En effet, Il est connu dans la littérature que les membres de la famille (comme la belle mère) ont une grande influence dans la prise de décision concernant la santé maternelle [20]. Prés de 60% des répondants préféraient l'accouchement à domicile. Les raisons économiques (gratuité) et géographiques (proximité) ont été rapportées. Ces motifs sont unanimement admis et représentent l'essentiel des déterminants du recours aux soins de santé. Le dernier accouchement n'était surveillé par un professionnel de santé que dans 26,2% des cas (N=55). La présentation de complications par la femme au moment de l'accouchement était le plus important motif du recours par le conjoint. En post-partum, 17% ont jugé qu'il n'est pas nécessaire d'effectuer la consultation post-natale et 36% méconnaissaient la possibilité de complications, ce dernier facteur était associé au non recours à la consultation post-natale par la conjointe (p = 0,021). Chez les 32% ayant eu recours aux structures de soins, la vaccination du nouveau-né était le motif principal (58,3%). Au fait, selon la réglementation marocaine, la délivrance du certificat de naissance est tributaire de la présentation du carnet de vaccination attestant avoir démarré le programme national de vaccination. Ce certificat est requis pour l'enregistrement du nouveau-né dans l'état civil. La perception des hommes interviewés met le point sur la persistance des barrières culturelles qui entravent l'implication effective des hommes. Les pratiques sont contradictoires avec leur prise de conscience de la nécessité de l'utilisation des soins en pré ou post-natal. Les freins d'ordre géographiques et socio-économiques ressortaient également lors de l'entretien, en plus de l'insatisfaction et le manque de confiance à l'égard des services de santé.

Le focus groupe (ou groupe centré) est une méthode pertinente pour l'étude des représentations sociales. Le couplage des deux méthodes qualitatives et quantitatives a offert une meilleure approche du problème de recherche et une plus profonde analyse des réponses de nos participants. L'autre avantage du présent travail est d'avoir touché à un sujet épineux dans une zone rurale qui rassemble plusieurs indicateurs de difficultés géo-climatiques et d'inaccessibilité aux soins. Ce qui pourrait servir d'exemple pour les intervenants dans le domaine surtout devant l'absence d'étude similaire. Nos résultats mettent le point sur les lacunes d'information et les besoins qui pourraient faire l'objet de compagnes de communication et de sensibilisation pour la promotion de la participation masculine dans la région aux programmes de régulation des naissances et de surveillance de la grossesse.

Notre échantillon était de faible taille vu les difficultés de recrutement et le caractère du sujet abordé encore considéré comme une affaire privée du couple dans notre société et surtout dans des zones reculées comme le site de la présente recherche. Mais la méthodologie adoptée permet d'obtenir - d'après l'expérience d'Immpact [12] - un échantillon représentatif de la population enquêtée. Certes il s'agit d'un échantillonnage non probabiliste et volontaire mais qui assure d'avoir un profil de recrus très proche du profil obtenu dans les enquêtes ayant couvert l'ensemble de la population. Ce qui répond à notre objectif d'approcher les représentations et les comportements.

## Conclusion

Au total, il paraît nécessaire de développer des actions de promotion de la santé, d'éducation de la population et d'information autour de la santé maternelle et reproductive. Les programmes de planification familiale et les visites en post-partum pourraient présenter une opportunité pour inclure le mari et le sensibiliser. Le changement des croyances et des comportements des hommes devraient être visé dans toutes les stratégies de réduction de la mortalité maternelle à travers des démarches participatives adaptées au contexte culturel. L'élargissement de la couverture médicale, le renforcement et l'amélioration des structures de soins pourraient également contribuer au changement du regard de la population et augmenter le recours à la surveillance de la grossesse et de l'accouchement.

### Etat des connaissances actuelle sur le sujet

- L'amélioration de la santé maternelle nécessite l'amélioration des connaissances et des comportements de la population, femmes et hommes;
- La perspective des hommes dans les dynamiques familiales est peu étudiée, et les recherches se concentrent sur les femmes;
- L'implication des hommes intéresse de plus en plus les chercheurs.

### Contribution de notre étude à la connaissance

- Décrire les connaissances, les attitudes et les comportements de l'homme rural marocain. Donner la parole aux hommes d'une région exemplaire par ses conditions de précarité;
- Mettre le point sur l'évolution des connaissances et des perceptions relatives à la santé reproductives, Mais en écart avec les pratiques des hommes;
- Une réflexion sur les moyens susceptibles de les impliquer d'avantage s'impose.
